# Why Don’t He Die?

**Published:** 1867-10

**Authors:** 


### WHY DON'T HE DIE?

"PIO NONO," according to newspaper writers, has been periodically on his last legs for at least half an age, and yet, although born in 1792, and has had a stormy reign, Pius the Ninth still lives, because he is a philosopher. In the first place, he has great benignity of disposition; writers agree in saying that on the very first instant the eyes light upon his features an indescribably winning effect is produced from the conviction of an inherent kindness of nature dwelling within. In the second place the venerable Pontiff has an extraordinary predilection for the greatest cleanliness of person, which is said to be next to godliness. In the third place, the simplicity of his diet is a model for all mankind. His breakfast is made of a piece of bread and a mixture of chocolate and coffee, at about 9 o'clock in the morning. He dines alone, takes a short nap, and takes a drive at 4 o'clock in the afternoon to the country, where he walks about for an hour and returns home at 6 o'clock and works about four hours, and goes to bed, thus not eating but twice a day. In summer-time the former Popes used to order refreshments of sherbets, ice-creams and various cooling drinks and pastries, but the Papal head takes a single orange, cuts it and squeezes it in a glass; and indeed there is nothing better to cool a person off in a warm day than an orange or a lemon. not only possessing considerable nutriment but containing an acid, which in its action on the general system is the very best antagonizer of fever. It is said that the "Holy Father' lives as simply and economically as when he was an obscure priest, that then one dollar a day supplied his table, and so it does now. The practical result of such an abstemious life is that "His Holiness," at the age of seventy-five years possesses an excellent constitution, is above the middle statue, has a full, broad chest and bids fair to live many a long day to come.

If any man or woman of forty-five or over, not engaged in hard manual labor, especially the studious, sedentary and in-door livers would take but two meals a day for one month, the second not being later than three in the afternoon, and absolutely nothing afterwards, except it might be in some cases an orange or lemon or cup of warm drink, such as tea, broma, sugar-water, or ice-cream, there would be such a change for the better in the way of sounder sleep, a feeling on waking of having rested, an appetite for breakfast, a bouyancy of disposition during the day, with a geniality of temper and manner that few, except the animal and the glutton, would be willing to go back to the flesh-pots of Egypt.

"Old Ben Wade," as he is called, eats but twice a day, is tough as leather and no doubt feels young enough to be the next President of the United States, although he has already reached his threescore and ten. Gourmandizing and gluttony are the bane of the age; half the people met on Broadway in any afternoon's walk are dyspeptic, and a large number of them have been so from childhood, brought on by the early drinking of tea and coffee, by premature dosing with soothing syrups, brandy toddy, and stuffing with cakes and other trash whenever a little hungry. At least ten years would be added to the average of human life in this country if a single rule were adopted for children's eating after ten years of age: To eat but three regular times every day, by sundown, and not an atom between meals.

---

"TWO MEALS A DAY." — "Ben Wade," as he is familiarly called, one of the political lions of the West, has taken but two meals a day for twenty years; and if all sedentary persons, those who are in-doors for the greater part of their time, would, after the age of forty-five, observe the same inflexible rule, there can be no doubt, other things being equal, that long years of happy exemption from the ordinary ills of life would be the result. The reason is, the stomach would have time to rest, for recuperation, and would thus be able to perform its part more thoroughly, making purer blood, giving better sleep, and securing a vigorous appetite for breakfast. Let any man try it for ten days, taking the second meal seven hours after the first, and abandon the practice if he can.

